# Analysis of Death Receptor 5 and Caspase-8 Expression in Primary and Metastatic Head and Neck Squamous Cell Carcinoma and Their Prognostic Impact

**DOI:** 10.1371/journal.pone.0012178

**Published:** 2010-08-16

**Authors:** Heath A. Elrod, Songqing Fan, Susan Muller, Georgia Z. Chen, Lin Pan, Mourad Tighiouart, Dong M. Shin, Fadlo R. Khuri, Shi-Yong Sun

**Affiliations:** 1 Department of Hematology and Medical Oncology, Emory University School of Medicine and Winship Cancer Institute, Atlanta, Georgia, United States of America; 2 Department of Pathology, Emory University School of Medicine and Winship Cancer Institute, Atlanta, Georgia, United States of America; 3 Department of Biostatistics and Bioinformatics, Emory University School of Medicine and Winship Cancer Institute, Atlanta, Georgia, United States of America; Karolinska Institutet, Sweden

## Abstract

Death receptor 5 (DR5) and caspase-8 are major components in the extrinsic apoptotic pathway. The alterations of the expression of these proteins during the metastasis of head and neck squamous cell carcinoma (HNSCC) and their prognostic impact have not been reported. The present study analyzes the expression of DR5 and caspase-8 by immunohistochemistry (IHC) in primary and metastatic HNSCCs and their impact on patient survival. Tumor samples in this study included 100 primary HNSCC with no evidence of metastasis, 100 primary HNSCC with lymph node metastasis (LNM) and 100 matching LNM. IHC analysis revealed a significant loss or downregulation of DR5 expression in primary tumors with metastasis and their matching LNM compared to primary tumors with no evidence of metastasis. A similar trend was observed in caspase-8 expression although it was not statistically significant. Downregulation of caspase-8 and DR5 expression was significantly correlated with poorly differentiated tumors compared to moderately and well differentiated tumors. Univariate analysis indicates that, in HNSCC with no metastasis, higher expression of caspase-8 significantly correlated with better disease-free survival and overall survival. However, in HNSCC with LNM, higher caspase-8 expression significantly correlated with poorer disease-free survival and overall survival. Similar results were also generated when we combined both DR5 and caspase-8. Taken together, we suggest that both DR5 and caspase-8 are involved in regulation of HNSCC metastasis. Our findings warrant further investigation on the dual role of caspase-8 in cancer development.

## Introduction

More than 35,000 people in the United States and more than 500,000 worldwide are estimated to be diagnosed with head and neck squamous cell carcinoma (HNSCC) annually [1], [2]. The presence of metastasis in patients with head and neck cancer is common and the 5-year survival rate for patients with lymph node metastasis is approximately 25–50% [3]. Better treatments for metastatic HNSCC are urgently needed. However, our understanding of the factors that regulate metastasis in this disease is limited.

Death receptor 5 (DR5) is one of the cell surface receptors that when activated by its ligand, tumor necrosis factor-related apoptosis-inducing ligand (TRAIL), induces the activation of the extrinsic apoptotic pathway in humans [4]. DR5 has been shown to be overexpressed in several types of cancer including colon, lung and cervical cancer [5]–[9]. Increased DR5 expression was also associated with reduced survival in non-small cell lung cancer [7], [9]. A recent mouse study has shown that deficiency of TRAIL receptor in mice (only one receptor for TRAIL in mouse) enhances lymph node metastasis (LNM) without affecting primary tumor development [10], suggesting that TRAIL receptor or TRAIL-TRAIL receptor interaction may be critical for regulation of tumor metastasis. Agonistic antibodies targeting DR5 are currently in clinical trials for treatment of various types of cancer [11]. Currently, the role of DR5 in metastasis is unknown and the expression of DR5 in primary and metastatic HNSCC has not been examined.

Caspase-8 is the first caspase activated during death receptor-initiated apoptosis [12]. There is evidence of increased expression of caspase-8 in several types of cancer including colorectal and rectal, gastric, pancreatic, and breast cancers [13], [14]–[17]. Besides, it has been also shown that caspase-8 expression is lost or inactivated in certain types of cancer such as small cell lung cancer, neuroblastoma, gastric carcinoma and hepatocellular carcinoma [18]–[25]. Loss of caspase-8 has been associated with metastasis in neuroblastoma [26]. However, it has also been recently shown that caspase-8 is associated with cell migration and can promote metastasis in apoptotic resistant cells [27], [28]. Moreover, a loss of caspase-8 was reported to be associated with unfavorable survival in childhood medulloblastoma [29]. Caspase-8 expression in HNSCC, particularly in metastatic HNSCC, has not been documented.

Thus, this study was particularly interested in comparing the expression patterns of DR5 and caspase-8 between primary HNSCC without LNM and HNSCC with LNM. To this end, we performed immunohistochemistry (IHC) to detect DR5 and caspase-8 on three groups of tumor samples from patients with either primary tumors with no evidence of LNM, primary tumors with LNM and the matching LNM.

## Materials and Methods

### Tissue Specimens

This study was approved by the Institutional Review Board at Emory University. Tissues were obtained from surgical specimens of patients who had HNSCC diagnosed at Emory University Hospital and whose initial treatment was surgery without receiving prior treatment with radiation and/or chemotherapy. The selection criteria of the available formalin-fixed and paraffin-embedded tissue blocks included 2 patient groups: primary HNSCC with LNM (Tu^+met^), their paired LNM, and primary HNSCC with negative LNM (Tu^−met^). In the Tu^−met^ group, if any patient developed metastases within 2 years of the initial procedure, they were excluded from the study. Each category has 100 samples. The clinical information on the samples was obtained from the surgical pathology files in the Department of Pathology at Emory University according to the regulations of the Health Insurance Portability and Accountability Act (HIPAA). This was a retrospective study which used tissue samples from surgical specimens dated prior to April 14, 2003 and therefore was exempt for consent requirement from HIPPA regulations. The clinicopathologic parameters for the 2 study groups, including age, gender, smoking history, tumor location, and histologic grade are listed in Table 1.

**Table 1:** Clinic-pathologic features of the non-metastatic and metastatic patient groups.

Clinical Parameters	Non-metastatic Group	Metastatic Group
**Average age** (years)	62.5	60.4
**Gender**		
Men	62	68
Women	41	33
**Smokers**	81*	91**
**Tumor location**		
Oral cavity	62	40
Oropharynx	7	33
Larynx	34	28
**Tumor classification**		
T1	42	24
T2	31	38
T3	14	17
T4	15	22
**Lymph node status**		
N1	-	19
N2	-	74
N3	-	8
**Histologic grade**		
WD	30	3
MD	60	75
PD	11	23

*Six patients with unknown smoking status.

**Four patients with unknown smoking status.

### IHC

Formalin-fixed, paraffin-embedded tissue sections were used for IHC. Tissues were deparaffinized, hydrated through graded ethanols, and microwaved in 100 mmol/L sodium citrate for 5 minutes at high power and 10 minutes at low power for antigen retrieval. Detection of caspase-8 and DR5 was performed following the DAKO Visualization System instructions using 3,3-diaminobenzidine tetrahydrochloride substrate to visualize the proteins (DAKO, Carpinteria, CA). The slides were incubated with caspase-8 polyclonal antibody (1∶100 dilution) (NeoMarkers, Fremont, CA) or DR5 polyclonal antibody (1∶250 dilution) (ProSci, Inc., Poway, CA) overnight at 4°C.

Both percentage of positive staining in tumor cells and intensity of staining were scored. The intensity of IHC staining was measured by using a numerical scale (0 = no expression, 1 = weak expression, 2 = moderate expression, 3 = strong expression). The staining data were finally quantified as the weighted index (WI) (WI  =  % positive stain in tumor × intensity score) as previously described [30], [31]. The WI was determined by 2 individuals, and the final values were the average of the two readings.

### Statistical Analysis

Median differences of the WIs for Caspase-8 and DR5 among different groups were assessed with Mann-Whitney-Wilcoxon rank test. Median differences between paired samples Tu^+met^ and LNM were analyzed with Wilcoxon signed rank test. Correlations of the WIs and the clinical characteristics for caspase-8 and DR5 were performed in all the patients and within each group and in the combined sample (Tu^−met^ and Tu^+met^) after adjusting with patients' metastatic status. Logistic regression model was applied to assess association between the WIs and binary variables (gender and smoking status). Kruskal-Wallis tests were performed for categorical variables with more than two categories (tumor site, tumor size, node status and differentiation status). The Cox proportional hazards model was used for univariate and multivariable survival analysis for continuous Caspase-8 and DR5. The proportional hazards assumption was assessed using Schoenfeld residuals. Caspase-8 and DR5 were also dichotomized as low and high based on the observed mean value. The log-rank test was used to test whether Kaplan-Meier survival estimators with different Caspase-8 or DR5 levels are statistically different. Multivariable analyses were performed with those clinical variables shown to be statistically significant in the univariate analyses. All data processing and statistical analyses were conducted using SAS version 9 (SAS Institute, Cary, NC).

## Results

### Detection of DR5 Expression in HNSCC

**Figure 1:**
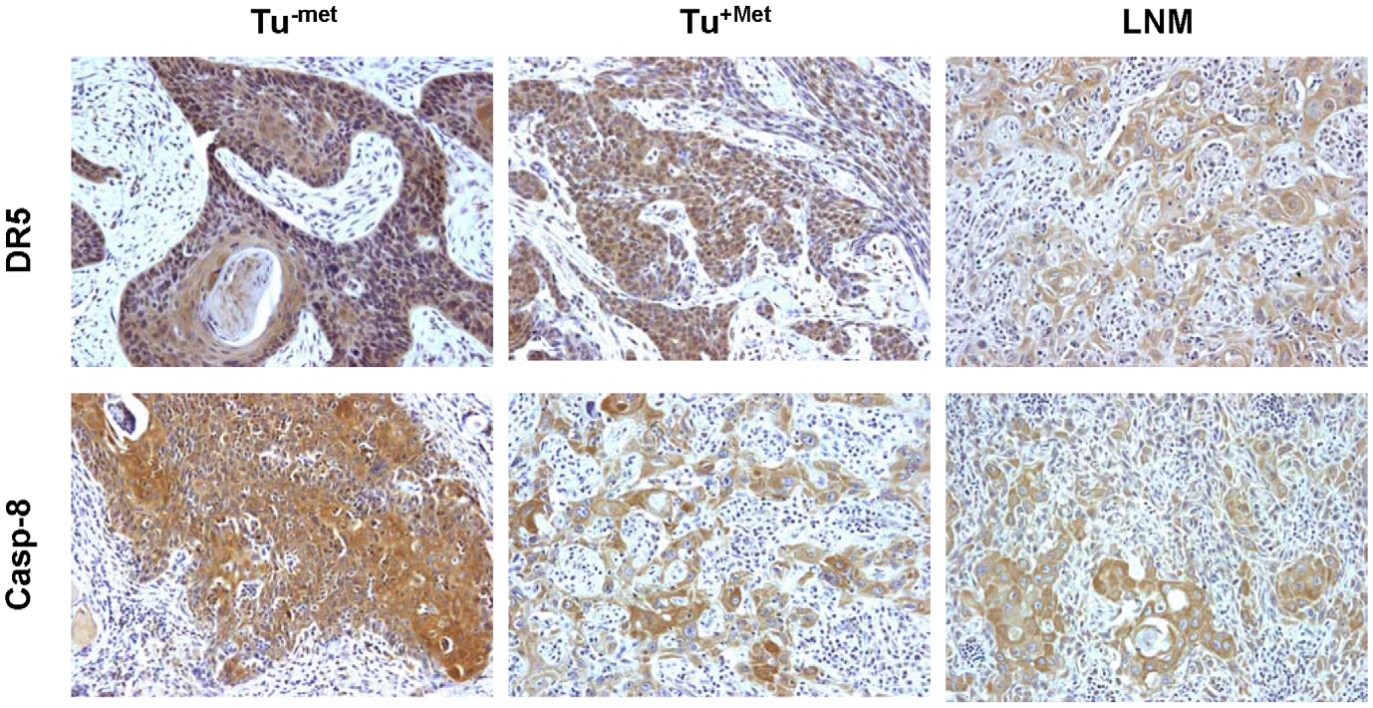
Representative IHC staining of DR5 and caspase-8 in different groups of HNSCC (200×).

**Figure 2:**
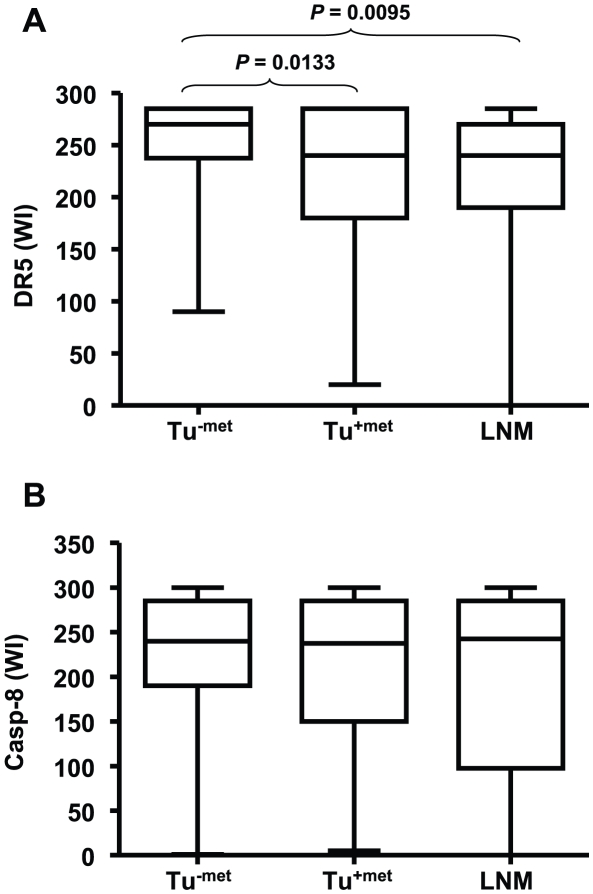
Comparison of DR5 (*A*) and caspase-8 (*B*) expression among different groups of HNSCC. Difference between two groups was evaluated with paired *t* test.

IHC analysis of DR5 was performed on 100 samples in each group. A total of 94 samples in Tu^−met^, 92 samples in Tu^+met^ and 85 samples in LNM group had acceptable tumor tissues for evaluation. All samples in Tu^−met^ and Tu^+met^ were positive for DR5 staining, whereas LNM tissues were 96% (82/85) positive for DR5 staining. Figure 1 shows examples of DR5 staining in different groups. DR5 was expressed primarily in the cytoplasm of tumor cells. Some tumor stromal cells including fibroblasts and immune cells were also positive for DR5. DR5 expression was often decreased or lost in Tu^+met^ and corresponding LNM samples. The analysis of the WI for DR5 showed that there was a statistically significant difference between DR5 expression in Tu^−met^ and in Tu^+met^ and their matching LNM samples (Figure 2A). Specifically, primary tumors without LNM (Tu^−met^) had significantly higher DR5 expression compared to both the primary tumors with LNM (Tu^+met^) and to LNM.

### Detection of Caspase-8 Expression in HNSCC

IHC analysis of caspase-8 was also conducted in 100 samples in each group. Among these samples, 96 Tu^−met^, 91 Tu^+met^ and 86 LNM samples could be evaluated. Some examples of caspase-8 staining were presented in Fig. 1. Similar to DR5 staining, caspase-8 staining was also primarily cytoplasmic. Some tumor stromal cells including fibroblasts and immune cells were positive for caspase-8. Tumors in Tu^−met^ group exhibited a trend towards a higher WI of caspase-8 than those in Tu^+met^ and LNM groups; however, this result was not statistically significant (Fig. 2B).

### DR5 Expression and its Correlation with Clinical Parameters

We further analyzed the correlation between DR5 expression and multiple clinical variables including gender, age at diagnosis, smoking status, tumor site, tumor size, tumor stage, histologic grade, node status, overall survival and disease free survival.

1. DR5 and tumor site. Univariate analysis showed that there was a significant difference in the location of the site of the tumor, characterized as oropharynx, larynx and oral cavity and the WI of DR5. Specifically, tumors in Tu^+met^ group arising in the oral cavity had significantly higher DR5 expression than tumors from this group that arose in the oropharynx or larynx (*P* = 0.0196).
2. DR5 and histologic grade. The histologic grade of the tumor samples were characterized as well differentiated (WD), moderately differentiated (MD) and poorly differentiated (PD). By univariate analysis, there was a significant correlation between histologic grade and DR5 expression. Primary tumors in Tu^+met^ and their matching LNM characterized as PD showed a significantly lower WI compared to MD and WD tumors in these groups (Figure 3A). Furthermore, multivariable analysis showed that in Tu^+met^ group, the PD tumors showed a significantly lower WI compared to tumors that were characterized as MD or WD (*P* = 0.0207). When combining all the patient tumor samples, univariate and multivariable analysis showed that histologic tumor grade was significantly correlated with DR5 expression. Specifically those tumors identified as PD have a lower DR5 WI compared to tumors identified as MD and WD. WD tumors have a higher WI compared to MD tumors. This result is consistent with analysis of the DR5 WI and histologic grade when comparing each group of tumors (Tu^+met^, LNM, and Tu^−met^) where we saw a decrease in DR5 expression as the tumors became less differentiated.

**Figure 3:**
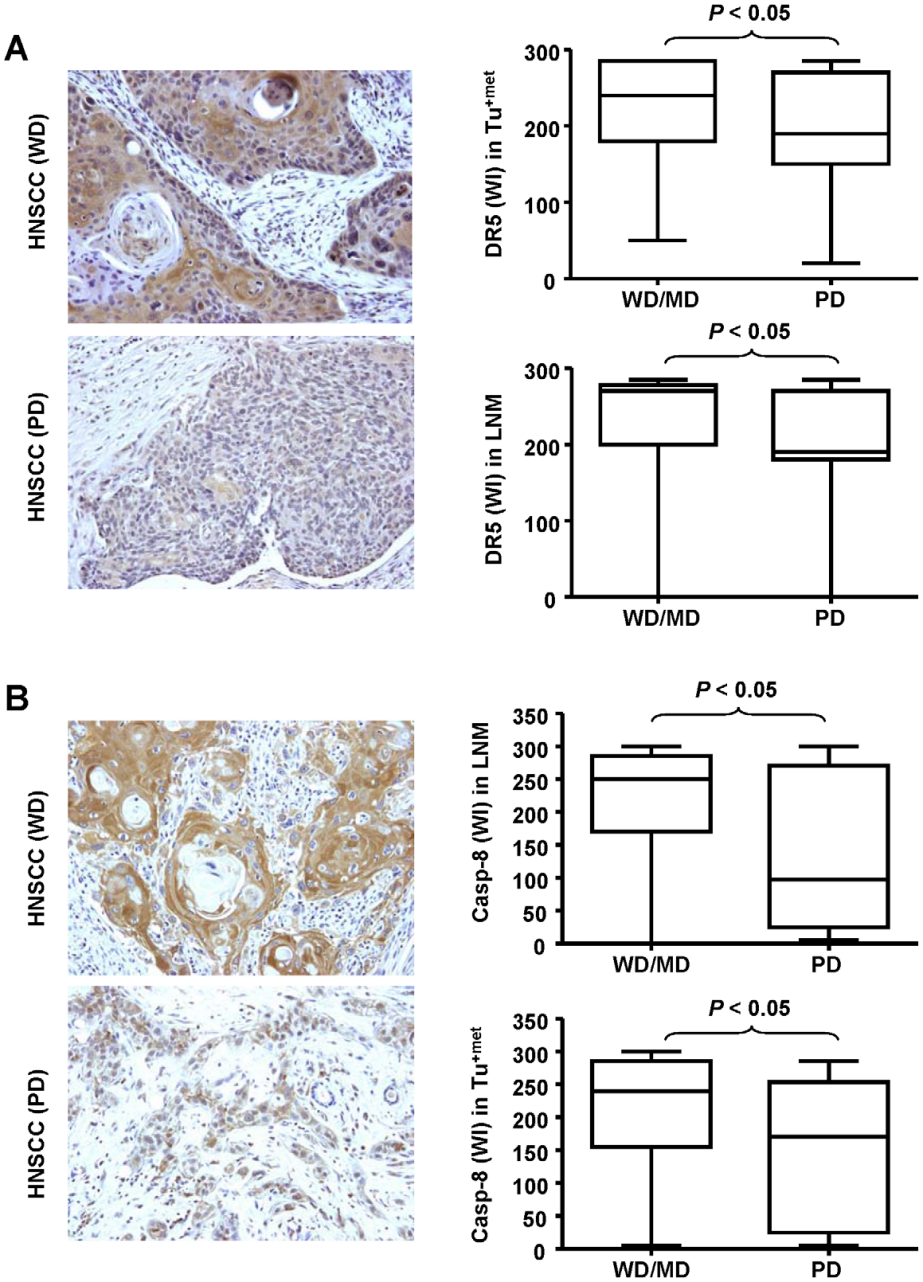
Comparison of DR5 (*A*) and caspase-8 (B) between poorly differentiated (PD) and well differentiated (WD)/moderate differentiated (MD) HNSCC. Pictures are representative IHC staining of DR5 and caspase-8 (200X). Difference between two groups was evaluated with paired *t* test.
3. DR5 and smoking status. Smoking status and DR5 expression was found to be significantly correlated when combining all tumor samples. By univariate and multivariable analysis, a lower DR5 WI was significantly associated with smokers compared to non-smokers when both groups of tumor samples were combined (i.e., Tu^−Met^ and Tu^+Met^) (*P*<0.05).
4. DR5 and patient survival. We analyzed whether DR5 expression has any effect on patient survival. Neither univariate nor multivariable Cox proportional hazards model revealed significant association between DR5 expression and patient survivals in either Tu^−met^ or Tu^+met^ group (*P*>0.05). However, when DR5 expression was dichotomized into low or high in terms of the observed mean value, higher DR5 expression was significantly associated with poorer disease-free survival (*P* = 0.0458) in Tu^+met^ group (Fig. 4B).

**Figure 4:**
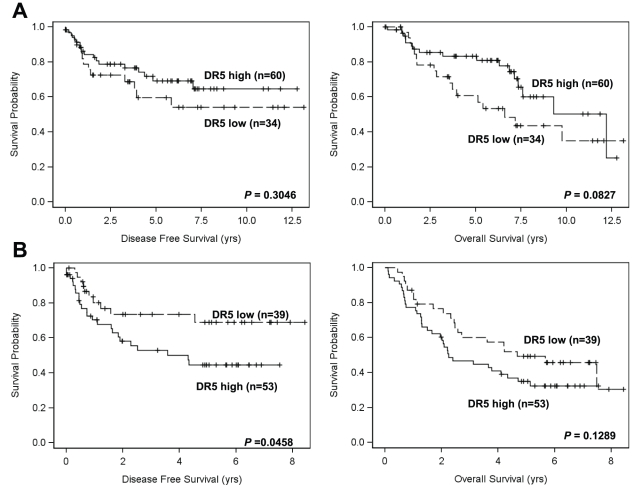
Impact of DR5 expression on disease-free survival and overall survival in HNSCC patients without LNM (Tu^−met^) (*A*) and in HNSCC patients with LNM (Tu^+met^) (*B*). Kaplan-Meier plots were generated according to high (greater than the mean value) and low (less than or equal to the mean value) levels of DR5.

### Caspase-8 Expression and Its Correlation with Clinical Parameters

Similar to DR5, an analysis of the correlation between caspase-8 expression and the clinical parameters including gender, age at diagnosis, smoking status, tumor site, tumor size, tumor stage, nodal status, histologic grade, overall survival and disease-free survival was performed.

**Figure 5:**
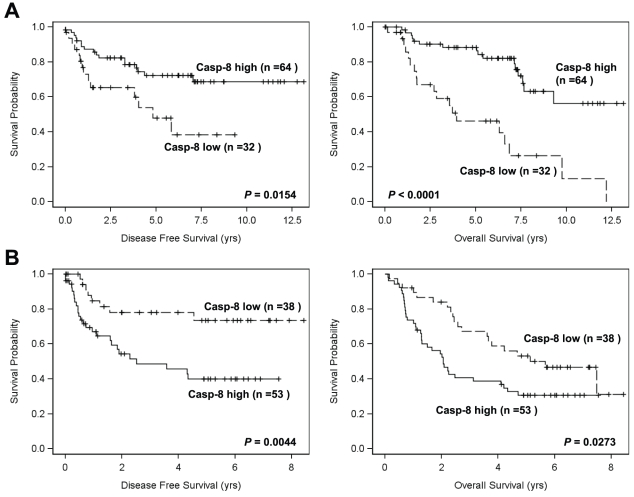
Impact of caspase-8 expression on disease-free survival and overall survival in HNSCC patients without LNM (Tu^−met^) (*A*) and in HNSCC patients with LNM (Tu^+met^) (*B* **).** Kaplan-Meier plots were generated according to high (greater than the mean value) and low (less than or equal to the mean value) levels of caspase-8.

1. Caspase-8 and tumor site. Univariate analysis showed that there was a significant difference in tumor location and the WI of caspase-8. Tumors in Tu^+met^ group arising in the oral cavity and their matching LNM had significantly higher caspase-8 expression than tumors that arose in the oropharynx or larynx (*P*<0.05).
2. Caspase-8 and histologic grade. By multivariable analysis, we found that caspase-8 expression was significantly reduced in PD primary tumors with metastasis (Tu^+met^) compared to the MD and WD tumors in this group (*P*<0.05). There were only three tumors characterized as WD in this group, so the WD and MD tumors were combined and their WI compared to the PD tumors. By univariate analysis, the PD tumors in the matching LNM had significantly less caspase-8 expression as measured by the WI compared to the combined MD and WD tumors in this group (Fig. 3B).
3. Caspase-8 and patient survival. We examined the impact of caspase-8 on patient survival and found that caspase-8 expression correlated significantly with disease-free survival and overall survival. Specifically, Cox proportional hazards model showed that in tumors with no metastasis (Tu^−met^), higher expression of caspase-8 was associated with better overall survival (*P* = 0.0053, HR = 0.994), while in tumors with LNM (Tu^+met^), higher caspase-8 expression was associated with poorer overall survival (*P* = 0.0347, HR = 1.003). The Log-rank test with dichotomized caspase-8 level showed the same effects with overall survival (Figs. 5A and 5B). In addition, the same effect was also observed with disease free survival (*P* = 0.0154 and 0.0044 in Tu^−met^ and Tu^−met^ group correspondingly) (Figs. 5A and 5B). Multivariable analysis adjusting for age, tumor stage, gender, histologic grade, smoking, chemo and/or radiation therapy, and tumor site showed that high caspase-8 (greater than mean) in the Tu^−met^ group was also significantly associated with better overall survival (*P* = 0.0026, HR = 0.255; see supplemental Table S1).

### Impact of DR5 and Caspase-8 Combination on Patient Survival

**Figure 6:**
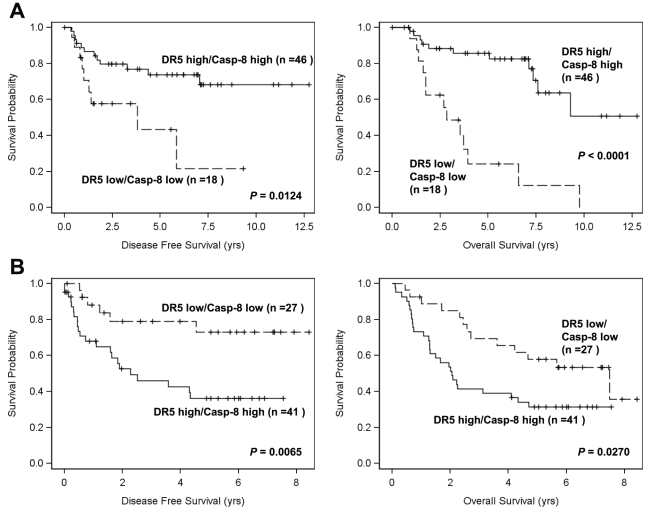
Impact of caspase-8 and DR5 combination on disease-free survival and overall survival in HNSCC patients without LNM (Tu^−met^) (*A*) and in HNSCC patients with LNM (Tu^+met^) (*B*). Kaplan-Meier plots were generated according to high (greater than the mean value) and low (less than or equal to the mean value) levels of DR5 and caspase-8.

Since both DR5 and caspase-8 are critical components in the extrinsic apoptotic pathway, we further analyzed the impact of DR5 and caspase-8 combination on HNSCC patient survival. The Log-rank test showed that the impact of the combined DR5 and caspase-8 on patient survival was identical to that of caspase-8 on patient survival. Specifically, in HNSCC with no LNM (Tu^−met^), patients with high levels of both DR5 and caspase-8 had better overall survival and disease-free survival relative to patients with low levels of both DR5 and caspase-8 (*P*<0.0001 and *P* = 0.0124, respectively) (Fig. 6A). In contrast, in HNSCC with LNM (Tu^+met^), patients with higher levels of both DR5 and caspase-8 had worse overall survival and disease-free survival relative to patients with low levels of both DR5 and caspase-8 (*P* = 0.0270 and *P* = 0.0065, respectively) (Fig. 6B).

## Discussion

The death receptor-mediated extrinsic apoptotic pathway plays an essential role in host immunosurveillance against tumor development, particularly metastasis [32]–[34]. In a genetic study, knockout of TRAIL receptor in mice does not affect primary tumor development, but enhances LNM [10], suggesting that TRAIL receptor is important for suppressing tumor metastasis. In human melanoma samples, a reduced DR5 expression was reported to be associated with metastatic lesions [35]. In agreement, the current study revealed a significant downregulation of DR5 expression in primary tumors with LNM (Tu^+met^) and their matching LNM compared to primary tumors with no metastasis (Tu^−met^). Moreover, DR5 expression was significantly reduced in PD tumors compared to MD and WD tumors. Therefore, our data on DR5 from human HNSCC samples supports an inhibitory role of DR5 in regulation of metastasis. The mechanism of DR5 in regulation of metastasis is not known. However, DR5 is involved in mediating anoikis, a form of apoptosis triggered by loss of attachment of cells from the extracellular matrix [36], [37]. Therefore, it is possible that the loss of DR5 we see in our metastatic HNSCC has contributed to the inhibition of anoikis, therefore allowing the tumor cells to escape apoptosis and migrate after detachment. It has been shown that in non-small cell lung carcinoma tissues, increased expression of DR5 correlates with PD tumors. Moreover, high DR5 expression is significantly associated with reduced overall survival [9]. However, our data clearly show that reduced DR5 expression correlates significantly with PD HNSCC. Furthermore, we did not find significant association between DR5 expression and overall survival or disease-free survival in Tu^−met^ group. In Tu^+met^ group, we found that the levels of DR5 were not associated with overall survival. However, higher DR5 was significantly associated with a worse disease-free survival. Despite the potential role of DR5 in negative regulation of metastasis as discussed above, our study did not show a survival advantage for Tu^+met^ HNSCC with high expression of DR5. In fact, one study has shown that TRAIL enhances the invasion of apoptosis-resistant pancreatic ductal adenocarcinoma cells *in vitro* and increases distant metastasis (e.g., liver) of pancreatic tumors *in vivo* [38], suggesting that activation of the DR5 signaling may facilitate metastasis under certain conditions. Moreover, the major mediator of DR5, caspase-8, has non-apoptotic functions that promote cell motility and migration as discussed below. Thus, whether DR5, like caspase-8, may also exert non-apoptotic functions, particularly in apoptotic resistant cells (see discussion below), should be further investigated.

We noted that DR5 staining in our study was primarily in the cytoplasm. However DR5 is known to be functional in inducing apoptosis as a membrane-bound protein. Given that DR5 expression levels do impact patient prognosis as demonstrated in this study, our data suggest that it may be interesting to study whether membrane-bound and cytoplasmic DR5 proteins exert distinct functions (e.g., apoptotic vs. non-apoptotic) under different conditions.

It has been shown that cigarette smoke impairs tumor immune surveillance and promotes invasion of cancer cells including oral carcinoma cells and tumor metastasis in experimental systems [39]–[43]. In this study, we found after examining all patient samples together that a lower DR5 expression in HNSCC was significantly associated with smokers compared to non-smokers. Thus, it would be interesting to determine if tobacco carcinogens can downregulate DR5 expression and possibly add to the effect of tumor cells escaping apoptosis or contributing to metastasis.

In addition to DR5, DR4 is another TRAIL receptor that can initiate death signaling upon TRAIL binding or overexpression [4]. Depending on tumor types, expression of DR4 has variable impact on prognosis. For example, high DR4 expression has been shown to be associated with worse disease-free survival, worse overall survival and shorter time to recurrence in colon cancer [6], whereas DR4 expression did not impact patient survival in lung, cervical and ovarian cancers [7], [44], [45]. Moreover, in breast cancer, DR4, in contrast to DR5, has been shown to be more strongly expressed in better differentiated tumors, and correlated positively with surrogate markers of a better prognosis (hormone receptor status, Bcl-2, negative nodal status), but negatively with the expression of Her2/neu and the proliferation marker Ki67 [46]. In our study, we did not stain DR4 expression in our cohort of HNSCC tissues, largely due to antibody issues. Nonetheless, it will be interesting to study DR4 expression in HNSCC and its association with LNM and prognosis in the future.

The major function of caspase-8 is to mediate apoptosis induced by death receptors including DR5. It has been recently suggested that caspase-8 can play dual roles: one as an inducer of apoptosis and one as a promoter in metastasis [47]. Caspase-8 facilitates cell death initiated from the death receptor pathway after a death ligand (e.g., TRAIL) binds a death receptor (e.g., DR5) [48]. In addition, caspase-8 can also promote cell migration [27], [49]–[51]. It has been suggested that caspase-8 can contribute to cell motility and adhesion by regulating calpain activity which controls cell migration including rac activation and lamellipodial assembly [50]. As well, the phosphorylation of procaspase-8 on tyrosine 380 and its interaction with the p85 alpha subunit of phosphatidylinositol 3-kinase was required to restore cell motility and adhesion in caspase-8 null cells [49]. However, there is evidence that a loss of caspase-8 is associated with increased metastasis. In neuroblastoma, a loss of caspase-8 prevented apoptosis by integrin-mediated cell death and therefore promoted metastasis [26]. In our study, caspase-8 expression trended towards a downregulation of expression in the metastatic group of patients, but this result was not statistically significant. Thus, it is unclear if the downregulation of caspase-8 that we observed in invasive HNSCC was playing a significant role in metastasis. It is possible that in primary tumors caspase-8 predominantly contributes to apoptosis and therefore can prevent metastasis, but in those tumor cells that escape apoptosis (i.e., are resistant to apoptosis), caspase-8 may be contributing to migration and metastasis. In our study, we found that in primary tumors with no LNM (i.e., Tu^−met^) higher expression of caspase-8 correlated with better disease-free survival and overall survival, however, in tumors with LNM (i.e., Tu^+met^) higher caspase-8 expression significantly correlated with worse disease-free survival and overall survival. Similar results were also generated when we analyzed the impact of caspase-8 and DR5 combination.Higher levels of both caspase-8 and DR5 in HNSCC with no LNM (Tu^−met^) was significantly associated with better disease-free survival and overall survival, but was significantly correlated with poorer disease-free survival and overall survival in HNSCC with LNM (Tu^+met^). Thus, it is plausible to speculate that caspase-8 (as well as DR5) in primary HNSCC without LNM (Tu^−met^) may be predominantly associated with its pro-apoptotic function and thus higher caspase-8 or caspase-8 plus DR5 provides protective advantage against cancer and correlates with better survival.Whereas in HNSCC with LNM (Tu^+met^) which are resistant to apoptosis, caspase-8 and even DR5 may primarily exert their non-apoptotic function, i.e., activation of PI3K and promotion of migration, and thus higher caspase-8 expression negatively impacts patient survival. A limitation of the current study is the existence of potential bias due to usage of selected patient populations and retrospective design although no tumor pretreatment was the major criteria for tumor selection. Nonetheless, our interesting findings warrant further study to demonstrate the precise role of caspase-8 as well as DR5/caspase-8 pathway in regulation of HNSCC metastasis.

In summary, our IHC analysis of caspase-8 and DR5 in HNSCC suggest that a loss or downregulation of DR5 expression and possibly caspase-8 expression may be associated with more metastatic tumors and a loss of differentiation. The overall high expression of DR5 and caspase-8 in both primary and metastatic HNSCC suggests that DR5 or caspase-8 may be a good target for therapy of HNSCC. Various agonistic DR5 antibodies such as Conatumumab (AMG655), CS-1008 and Lexatumumab have been currently tested in cancer clinical trials either as a single agent or in combination with other therapeutic agents. Early clinical trials of these agents have established the safety of the approach and showed proof-of-concept antitumor activity [52]. Thus, our current findings warrant further study on targeting DR5 for potential treatment of HNSCC.

## Supporting Information

Table S1 (0.04 MB DOC)
