# Supplementary material for: Analysis of Death Receptor 5 and Caspase-8 Expression in Primary and Metastatic Head and Neck Squamous Cell Carcinoma and Their Prognostic Impact
Source: PLoS One. 2010 Aug 16;5(8):e12178. doi: 10.1371/journal.pone.0012178 (PMC2922336; doi:10.1371/journal.pone.0012178)
Supplement: Table S1 — (0.04 MB DOC) [file pone.0012178.s001.doc]

Table S1. Multivariable analyses of overall survival and Caspase-8 in Tu^-Met^ patients

| *Analysis of Maximum Likelihood Estimates* | | | | | | | |
| --- | --- | --- | --- | --- | --- | --- | --- |
| *Parameter* | *DF* | *Parameter Estimate* | *Standard Error* | *Chi-Square* | *Pr > ChiSq* | *Hazard Ratio* | *Label* |
| *Cas-8 Tu^-Met^-mean* | 1 | -1.36791 | 0.45336 | 9.1041 | 0.0026 | 0.255 |  |
| *Age* | 1 | 0.04813 | 0.01711 | 7.9163 | 0.0049 | 1.049 | Age |
| *Gender* | 1 | 0.53984 | 0.53484 | 1.0188 | 0.3128 | 1.716 |  |
| *Tumor stage* | 1 | 0.31194 | 0.18825 | 2.7457 | 0.0975 | 1.366 | T |
| *Histologic grade* | 1 | 0.01861 | 0.21570 | 0.0074 | 0.9312 | 1.019 |  |
| *Smoking* | 1 | -1.06973 | 0.59444 | 3.2384 | 0.0719 | 0.343 |  |
| *Chemotherapy* | 1 | 0.77935 | 0.69361 | 1.2625 | 0.2612 | 2.180 |  |
| *Radiation therapy* | 1 | 0.56413 | 0.45244 | 1.5546 | 0.2125 | 1.758 |  |
| *Tumor site* | 1 | 0.51752 | 0.32292 | 2.5684 | 0.1090 | 1.678 |  |
